# Natural Clinopyroxene Reference Materials for *in situ* Sr Isotopic Analysis via LA-MC-ICP-MS

**DOI:** 10.3389/fchem.2020.594316

**Published:** 2020-12-09

**Authors:** Han Zhao, Xin-Miao Zhao, P. J. Le Roux, Wen Zhang, Hao Wang, Lie-Wen Xie, Chao Huang, Shi-Tou Wu, Jin-Hui Yang, Fu-Yuan Wu, Yue-Heng Yang

**Affiliations:** ^1^State Key Laboratory of Lithospheric Evolution, Institute of Geology and Geophysics, Chinese Academy of Sciences, Beijing, China; ^2^Innovation Academy of Earth Science, Chinese Academy of Sciences, Beijing, China; ^3^College of Earth and Planetary Sciences, University of Chinese Academy of Sciences, Beijing, China; ^4^Department of Geological Sciences, University of Cape Town, Cape Town, South Africa; ^5^State Key Laboratory of Geological Processes and Mineral Resources, China University of Geosciences, Wuhan, China

**Keywords:** clinopyroxene, *in situ*, ^87^Sr/^86^Sr analysis, LA-MC-ICP-MS, natural reference material

## Abstract

Clinopyroxene is a major host mineral for lithophile elements in the mantle lithosphere, and therefore, its origin is vital for constraints on mantle evolution and melt generation. *In situ* Sr isotopic measurement of clinopyroxene has been available since the recent development of laser ablation multicollector inductively coupled plasma mass spectrometry (LA-MC-ICP-MS) in the 2000s. Therefore, there is an increasing demand for natural clinopyroxene reference materials for Sr isotope microanalysis. In this contribution, we present six natural clinopyroxene reference materials from South Africa (JJG1424) and China (YY09-47, YY09-04, YY09-24, YY12-01, and YY12-02) for Sr isotope microanalysis. The Sr content of these clinopyroxenes ranges from 50 to 340 μg g^−1^, which covers most natural clinopyroxene compositions. Homogeneity of these potential reference materials were investigated and evaluated in detail over a 2-year period using 193-nm nanosecond and 257-nm femtosecond laser systems coupled to either a Neptune or Neptune Plus MC-ICP-MS. Additionally, the major and trace element of these clinopyroxenes were examined by electron probe microanalyzer (EPMA) as well as solution and laser ICP-MS. The *in situ* ^87^Sr/^86^Sr values obtained for the six natural clinopyroxene reference materials agree well with data obtained using the thermal ionization mass spectrometer (TIMS) method. The Sr isotopic stability and homogeneity of these clinopyroxenes make them potential reference materials for *in situ* Sr microanalysis to correct instrumental fractionation or as quality control materials for analytical sessions. The new Sr isotope data provided here might be beneficial for microbeam analysis in the geochemical community.

## Highlights

- Six natural clinopyroxene reference materials available for *in situ* Sr isotope analysis.
- The Sr content covers most natural clinopyroxene compositions.
- Homogeneous investigation using nano- and femtosecond LA-MC-ICP-MS.
- *In situ* ^87^Sr/^86^Sr values in good agreement with those data by TIMS method.

## Introduction

Clinopyroxene (CPX), a common Ca-rich mineral in mantle rock, is a principal host of Sr, and its Sr isotopic composition can reveal geological processes in the mantle lithosphere, such as (1) melt refertilization of the mantle (Tang et al., 2011, 2012; Zou et al., 2014; Liu et al., 2016); (2) degree of partial melting the mantle experienced (Griffin and Brueckner, 1985; Norman, 2001; Liu et al., 2016); and (3) metasomatic agents affecting the mantle (Rudnick et al., 2004; Wu et al., 2006; Sapienza et al., 2008; Touron et al., 2008; Sun et al., 2012; Xu et al., 2013; Tang et al., 2017; Aulbach et al., 2019). In summary, clinopyroxene Sr isotopes may therefore be a powerful tracer of subducted altered oceanic crust (Xu et al., 2003; Gao and Zhou, 2013).

*In situ* Sr isotope measurement via ablation multicollector inductively coupled plasma mass spectrometry (LA-MC-ICP-MS) for clinopyroxene has been growing rapidly. For example, the first study demonstrated isotopic heterogeneity at the scale of individual grains in peridotite xenoliths and multiple measurements of the same grain; however, it indicated intragrain Sr isotopic disequilibrium (Schmidberger et al., 2003). Subsequently, Jackson and Hart (2006) reported *in situ* Sr isotopes in melt inclusions hosted by olivine phenocrysts and observed melts from high ^3^He/^4^He and EM II-type mantle end members, respectively. Sun et al. (2012) performed an *in situ* Sr isotopes for clinopyroxene in mantle xenoliths from Hebi, central North China Craton (NCC), and concluded that the clinopyroxene were crystallized from metasomatic melts. Xu et al. (2013) conducted *in situ* Sr isotopic composition of peridotite xenoliths from Kuandian and investigated Pacific slab subduction-related mantle modification of clinopyroxene beneath the eastern NCC.

Although solution-based thermal ionization mass spectrometer (TIMS) or MC-ICP-MS method, in particular, is suitable for high precision Sr isotope determination, after laborious and time-consuming chemical purification (Chu et al., 2009; Yang et al., 2010, 2011, 2012; Li et al., 2012; Raddatz et al., 2013; Xu et al., 2020), LA-MC-ICP-MS is extensively applied to analyze Sr isotope on account of its rapid sample preparation, high throughput, and small volume consumption (Schmidberger et al., 2003, 2007; Jackson and Hart, 2006; Fietzke et al., 2008; Konter and Storm, 2014). Moreover, *in situ* Sr isotopic analysis can decipher more significant information than common whole rock analysis in high resolution. Currently, LA-MC-ICP-MS is a well-developed technique to measure ^87^Sr/^86^Sr in relatively high Sr geological samples (>500 μg g^−1^ Sr, e.g., apatite, perovskite, plagioclase, eudialyte, bastnaesite) (Yang et al., 2009a, 2011, 2019; Wu et al., 2010; Kimura et al., 2013; Konter and Storm, 2014; Tong et al., 2016; Zhang et al., 2018). Nevertheless, for low Sr-content minerals, such as clinopyroxene from most peridotite xenoliths, it is still challenging (Waight et al., 2002; Woodhead et al., 2005; Copeland et al., 2008; Vroon et al., 2008; Jochum et al., 2009; Lin et al., 2015).

**Figure 1:**
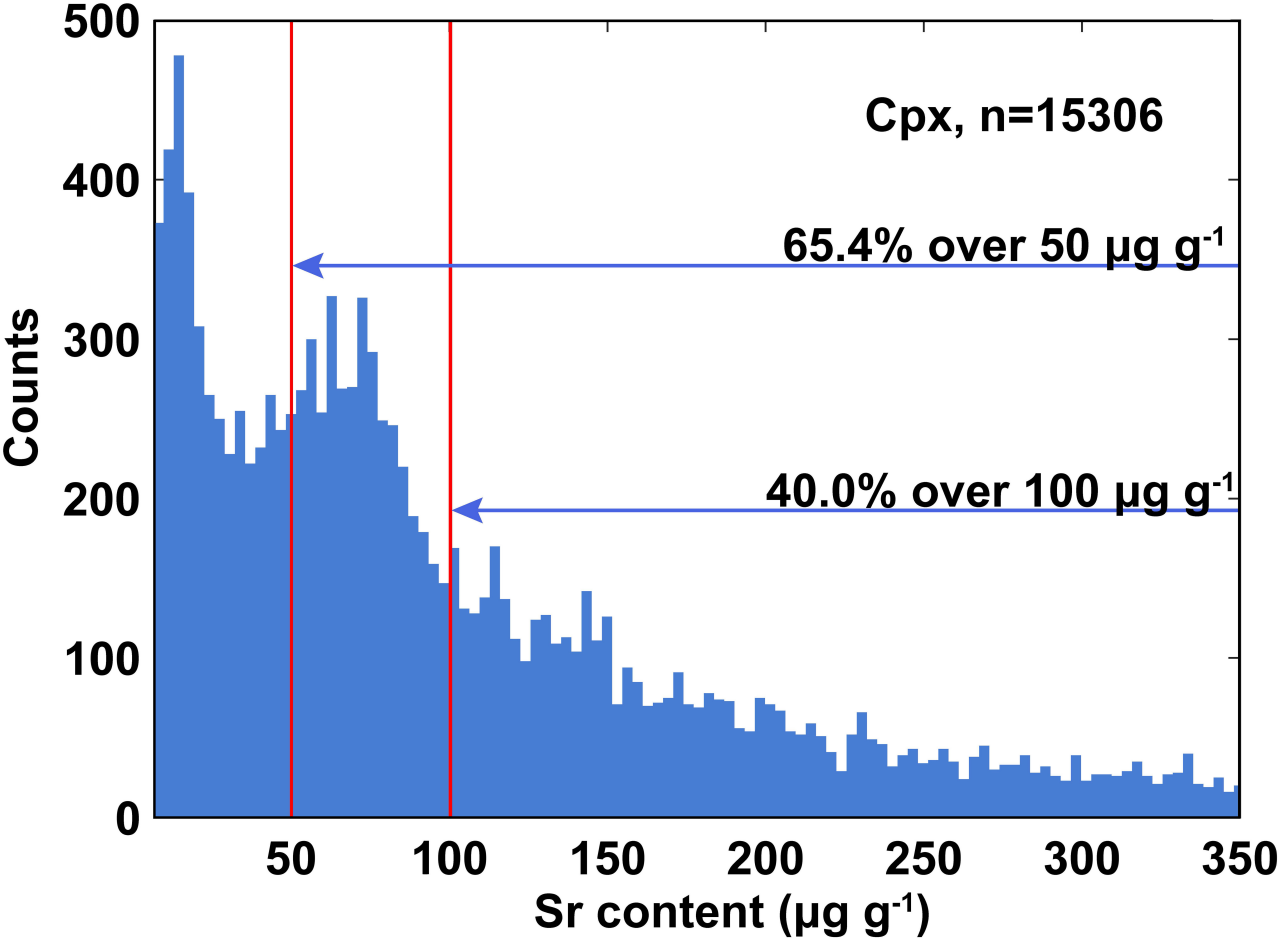
Sr contents (μg g^−1^) distribution range over 10,000 published clinopyroxenes (based on data from Georoc).

According to the literature data from Georoc (Figure 1), the Sr content of natural clinopyroxene samples mainly distributed between 50 and 350 μg g^−1^. Although a synthesized clinopyroxene glass with added Sr (CPX05G, ~518 μg g^−1^ Sr) was developed as an in-house reference material, the content of Sr is higher than that of most natural clinopyroxene, and a limitation is its unavailability for other users (Tong et al., 2016). There is still lack of accessible clinopyroxene reference material for Sr isotope microanalysis. Despite a few published papers about *in situ* Sr analysis of clinopyroxene, the shortage of reference materials hinders the development of *in situ* Sr isotope measurements for clinopyroxene (Waight et al., 2002; Bizzarro et al., 2003; Schmidberger et al., 2003; Hart et al., 2005; Sun et al., 2012; Xu et al., 2013; Su et al., 2015; Tong et al., 2016; Deng et al., 2017; Tang et al., 2019).

Herein, we investigated whether the homogeneity of Sr isotopes for six natural clinopyroxene from South Africa (JJG1424) and China (YY09-47, YY09-04, YY09-24, YY12-01, and YY12-02), which covers a range of 50–350 μg g^−1^, corresponds well to the natural distribution, at the micrometer scale, using MC-ICP-MS coupled with nano- and femtosecond laser over a 2-year period. Precise Sr isotope compositions of these samples were also determined using classic TIMS or solution MC-ICP-MS methods. Meanwhile, the major and trace element of these clinopyroxenes were examined by EPMA, ICP-MS based on solution, and laser sampling. Our work indicates that these six natural minerals might be employed as potential reference materials for *in situ* Sr isotope analysis.

## Analytical Methods

All six samples were prepared and mounted in epoxy resin blocks and polished to expose the interior of the crystals prior to analysis at the State Key Laboratory of Lithospheric Evolution (SKLLE), Institute of Geology and Geophysics, Chinese Academy of Sciences (IGGCAS). For comparison, one analytical session for *in situ* Sr isotope was performed at the State Key Laboratory of Geological Process and Mineral Resources (GPMR), China University of Geosciences (Wuhan), China.

### Sample Description

Five of the samples were separated from lherzolite xenoliths entrained in the Yangyuan, in the Central Zone of the NCC (Zhao et al., 2015, 2017), and the other is from South Africa (Class and le Roex, 2011). According to the mineralogical and geochemical features of the clinopyroxene, they are Cr-diopside.

### Major Element by EPMA

Major element composition of samples and backscattered electron (BSE) images were obtained from polished thin sections using a JEOL-JXA8100 electron probe microanalyzer (EPMA) at IGGCAS. The operating conditions were as follows: 15 kV accelerating voltage, 12 nA beam current, 5 μm beam spot, and 10–30 s counting time on peak. Natural clinopyroxene and synthetic oxides were used for data correction, and the precision of all analyzed elements is better than 1.5%.

### Trace Element Compositions Using Solution or Laser Ablation ICP-MS Analysis

Solution trace element contents in clinopyroxene were determined using a sector field (SF) ICP-MS (Finnigan MAT Element I) after digestion of about 40 mg of sample using a mixture of ultrapure 1 ml HF and 0.8 ml HNO_3_ in Teflon bombs. After dissolution, the solution in the bomb is transferred into a polyethylene terephthalate (PET) bottle, which is weighed accurately to 50 g by addition of a 2% HNO_3_ solution with 10 ng g^−1^ in internal standard addition. The carrier and makeup gas flows were optimized daily to obtain a sensitivity of ^89^Y over 20 Mcps/μg g^−1^ while holding the ThO^+^/Th^+^ ratio below 0.5%. Indium was used as an internal standard to correct for matrix effects and instrumental drift. A Chinese GSR-3 silicate reference material was measured to monitor the accuracy of the analytical procedure, and the results are in consistence with recommended values. The results are adopted as reference values for LA-ICP-MS analyses. According to the result of GSR-3, individual elemental precision is generally better than 5%.

An Agilent 7500a ICP-MS coupled with a 193-nm ArF excimer laser ablation system was employed to measure trace element. Helium was used as the carrier gas through the ablation cell and mixed with argon downstream of the ablation cell (Table 1). Prior to measurement, the pulse/analog (P/A) factor of the detector was calibrated using a tuning solution. The carrier and makeup gas flows were daily optimized to obtain maximum signal intensity for ^238^U^+^ while keeping the ThO^+^/Th^+^ ratio below 0.5%. All LA-ICP-MS determinations were conducted using time-resolved analysis in fast, peak jumping mode. Each spot analysis consisted of an ~20-s background and 60-s sample data acquisition. The dwell time for each isotope was set at 6 ms for Rb, Sr, Ba, Nb, Ta, Zr, Hf, Pb, and rare earth element (REE) and 10 ms for ^232^Th and ^238^U. Trace element concentrations were calibrated against the NIST SRM 612 standard glass reference material with ^43^Ca as the internal standard element and using USGS BCR-2G glass as a quality monitor. Data reduction, including concentration determinations, method detection limits, and internal uncertainties were obtained using the GLITTER laser ablation software (Achterbergh et al., 2001).

Table 1Typical instrumental parameters for trace element and Sr isotopic measurements by LA-(MC)-ICP-MS.

**Table:** 

**Laser ablation systems**	**Coherent Geolas Plus and Analyte G2**	**New wave research**
Laser system	ComPex 102, ArF excimer UV 193 nm ATLex 300si, ArF excimer UV 193 nm	Yb:YAG femtosecond laser 257 nm
Ablation cell and volume	Standard circle low volume cell, volume ca. 4 cm^3^ Commercial HeLEx dual-volume cell	Commercial Two-Volume Chamber
Fluence	~ 5 J/cm^2^ for trace element, ~ 8 J/cm^2^ for Sr isotope	0.50–3.85 J/cm^2^
Repetition rate	8 Hz	1–1,000 Hz
Spot diameter nominal	60 μm for trace elements, 60~120 μm for Sr isotope	45, 60 μm
Ablation duration	60 s for trace elements, 60 s for Sr isotope	60 s for Sr isotope
Sampling mode	Static spot ablation	Line scanning (5 μm s^−1^)
Sample preparation	Conventional mineral separation, 1 inch resin mount

**Table:** 

**Mass spectrometers**	Neptune or Neptune Plus MC-ICP-MS	Agilent 7500a Q-ICP-MS	
RF forward power (W)	~1,300	RF forward power (W)	~1,350
Cool gas (L/min)	16	Carrier gas (L/min)	~1.1
Auxiliary gas (L/min)	0.8	Sample depth (mm)	~4.5
Carrier gas flow (L/min)	~1.2	Interface cone	Ni
Sampling cone	Ni, aperture 1.0 mm	Dwell times	6 ms for trace elements 90 s (including 30 s background, 60 s ablation)
Skimmer cone	Ni, aperture 0.8 mm		
Sampling mode	1 block of 200 cycles		
Integration time	0.524s		
Background/baseline	30 s on peak zero (OPZ)

**Table:** 

**MC-ICP-MS cup configuration**									
Faraday cups	L4	L3	L2	L1	Center	H1	H2	H3	H4
Mass	83	83.5	84	85	86	86.5	87	88	89
Sr^+^			^84^Sr^+^		^86^Sr^+^		^87^Sr^+^	^88^Sr^+^	
Kr^+^	^83^Kr^+^		^84^Kr^+^	^85^Rb^+^			^87^Rb^+^		
[CaAr]^+^	[^43^Ca^40^Ar] ^+^ [^43^Ca^40^Ca] ^+^		[^44^Ca^40^Ar] ^+^ [^44^Ca^42^Ca] ^+^ [^42^Ca^42^Ca] ^+^	[^43^Ca^42^Ca] ^+^	[^46^Ca^40^Ar] ^+^ [^48^Ca^38^Ar] ^+^ [^46^Ca^40^Ca] ^+^ [^44^Ca^42^Ca] ^+^ [^43^Ca^43^Ca] ^+^		[^44^Ca^43^Ca] ^+^	[^48^Ca^40^Ar] ^+^ [^48^Ca^40^Ca] ^+^ [^46^Ca^42^Ca] ^+^ [^44^Ca^44^Ca] ^+^	
REE^++^	^166^Er^++^	^167^Er^++^	^168^Er^++^	^170^Er^++^ ^170^Yb^++^	^172^Yb^++^	^173^Yb^++^	^174^Yb^++^ ^174^Hf^++^	^176^Yb^++^ ^176^Hf^++^	Y^+^

**Table:** 

**Data processing**	
Gas blank	30 s on-peak zero subtracted
Calibration strategy	NIST 612 used as external standard and ^43^Ca used as internal standard for calibrating trace elements, ^83^Kr/^84^Kr = 0.20175, ^83^Kr/^86^Kr = 0.66474, ^85^Rb/^87^Rb = 2.5926; (Christensen et al., 1995; Bizzarro et al., 2003; Woodhead et al., 2005)
Data processing package used	For trace elements, Glitter software was used for isotopic and elemental fractionation, instrumental mass bias calibration and uncertainty propagation. For Sr isotope, an in house Microsoft Excel macro written in VBA (Visual Basic for Applications) was used for Sr isotope mass fraction correction, interference correction and uncertainty propagation.

### *In situ* Sr Isotopic Analysis by Laser Ablation MC-ICP-MS

Neptune or Neptune Plus MC-ICP-MS with either a 193-nm ArF excimer or 257-nm femtosecond laser was employed to measure Sr isotopes at IGGCAS, Beijing and GPMR, Wuhan (Yang et al., 2014, 2019; Zhang et al., 2018, 2020). A spot size of 60–160 μm was used with a 6–8 Hz repetition rate and an energy density of ~8 J cm^−2^, depending on the Sr content of sample (Table 1). The Sr isotopic data were collected by static multicollection mode, using X skimmer and Jet sample cone. Prior to laser measurement, the MC-ICP-MS was optimized using a standard solution to obtain maximum sensitivity. The integral process of data acquisition has one block of 200 cycles, and the integration time is 0.524 s per cycle. A typical data acquisition cycle consisted of a 30-s measurement of the Kr gas blank with the laser off, followed by 60 s of measurement with the laser on. In this work, YY09-47, YY09-24, and YY12-01 clinopyroxene samples were measured after every 10 unknown samples for external calibration (Yang et al., 2014, 2019; Lin et al., 2016; Zhang et al., 2018, 2020).

Data reduction was conducted offline and the potential isobaric interferences were accounted for in the following order: Kr^+^ and Rb^+^. First, the interference of ^84^Kr and ^86^Kr on ^84^Sr and ^86^Sr, respectively, were removed using the 30-s Kr gas baseline measurement. The isobaric interference correction of ^84^Kr and ^86^Kr on ^84^Sr and ^86^Sr was carried out using the natural Kr isotopic ratios (^83^Kr/^84^Kr = 0.20175, ^83^Kr/^86^Kr = 0.66474; Christensen et al., 1995; Bizzarro et al., 2003). Second, the natural ratio of ^85^Rb/^87^Rb (2.5926) was used to correct for isobaric interference of ^87^Rb on ^87^Sr by the exponential law, assuming that Rb has the same mass discrimination behavior as Sr (Woodhead et al., 2005). It is observed that the obtained ^87^Rb/^87^Sr ratio is typically <0.001 during *in situ* clinopyroxene Sr analysis, indicating that the radiogenic ^87^Sr contribution is negligible (Yang et al., 2011). Additionally, our previous work demonstrated that Ca argides and dimers had an insignificant influence on Sr isotope analysis using a Neptune MC-ICP-MS (Yang et al., 2011); this observation is also strongly supported by other studies (Ramos et al., 2004; Vroon et al., 2008). Therefore, interferences from Ca argides or dimers are not considered further in this work. Meanwhile, we also monitored the ^167^Er^2+^, ^171^Yb^2+^, and ^173^Yb^2+^ at masses 83.5, 85.5, and 86.5, indicating negligible interference of double-charged ion. Finally, the ^87^Sr/^86^Sr ratios were calculated and normalized from the interference-corrected ^86^Sr/^88^Sr ratio using the exponential law. The whole data reduction procedure was performed using an in-house Excel Visual Basic for Applications (VBA) macro program (Horstwood et al., 2008, 2016; Zhang et al., 2018; Zhang et al., 2020).

### Solution Sr Isotope Measurement by Isotope Dilution Thermal Ionization Mass Spectrometer

Rb and Sr concentration and isotopic compositions of the clinopyroxenes were measured using a Thermo Scientific Triton Plus TIMS in IGGCAS. Clinopyroxenes from a number of samples were handpicked for radiogenic isotope analysis using the isotope dilution method detailed elsewhere (Li et al., 2012, 2019). About 50 mg of each sample was weighted into a 7-ml round bottom Savillex™ Teflon screw-top capsule and 3.0 ml of a mixed acid added, composed of 29 M HF + 0.3 ml 14 M HNO_3_ + 0.3 ml 11.8 M HClO_4_ with the addition of a ^87^Rb–^84^Sr tracer. The samples were dissolved on a hotplate at 180°C for 7 days. Each capsule was opened and evaporated to fume HClO_4_ after cooling. The dissolved sample solution was then evaporated to dryness at ca. 120°C. After that, the samples were redissolved once more in 1.0 ml of 6 M HCl and reheated to 180°C for several hours to eliminate fluoride complexes. Finally, the vials were opened, and the resulting sample solution was evaporated to dryness and redissolved with 1.0 ml of 2.5 M HCl on a hot plate at 120°C. Next, elements were separated on AG50W-X12 cation resin columns (Li et al., 2012, 2019). Rock reference materials BCR-2 and BHVO-2 from USGS were measured to monitor the accuracy of the analytical procedure, and our results [BCR-2: 46.2 μg g^−1^ Rb and 338 μg g^−1^ Sr, 0.705004 ± 0.000008 (2μ) of ^87^Sr/^86^Sr ratio; BHVO-2: 8.9 μg g^−1^ Rb and 378 μg g^−1^ Sr, 0.703419 ± 0.0000013 (2μ) of ^87^Sr/^86^Sr ratio] are almost identical to recommended values (Raczek et al., 2003; Yang et al., 2010, 2012).

## Results and Discussion

**Figure 2:**
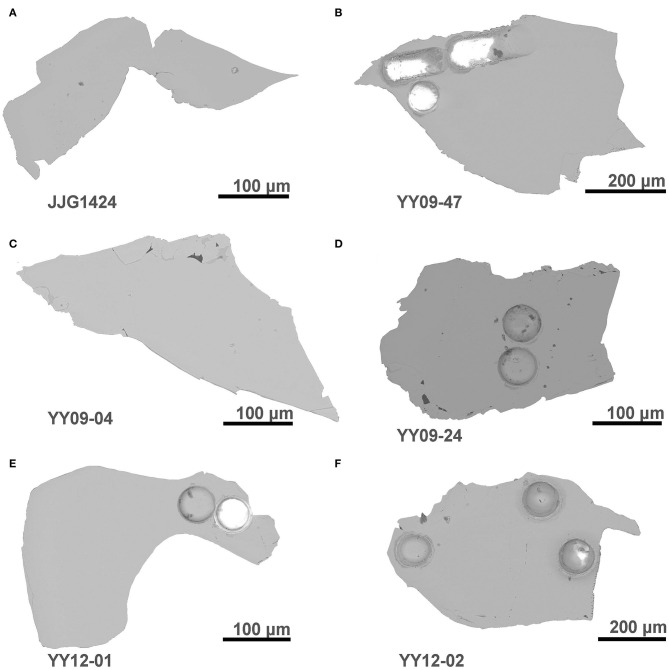
Backscattered images of representative grains of clinopyroxene investigated in this work.

**Figure 3:**
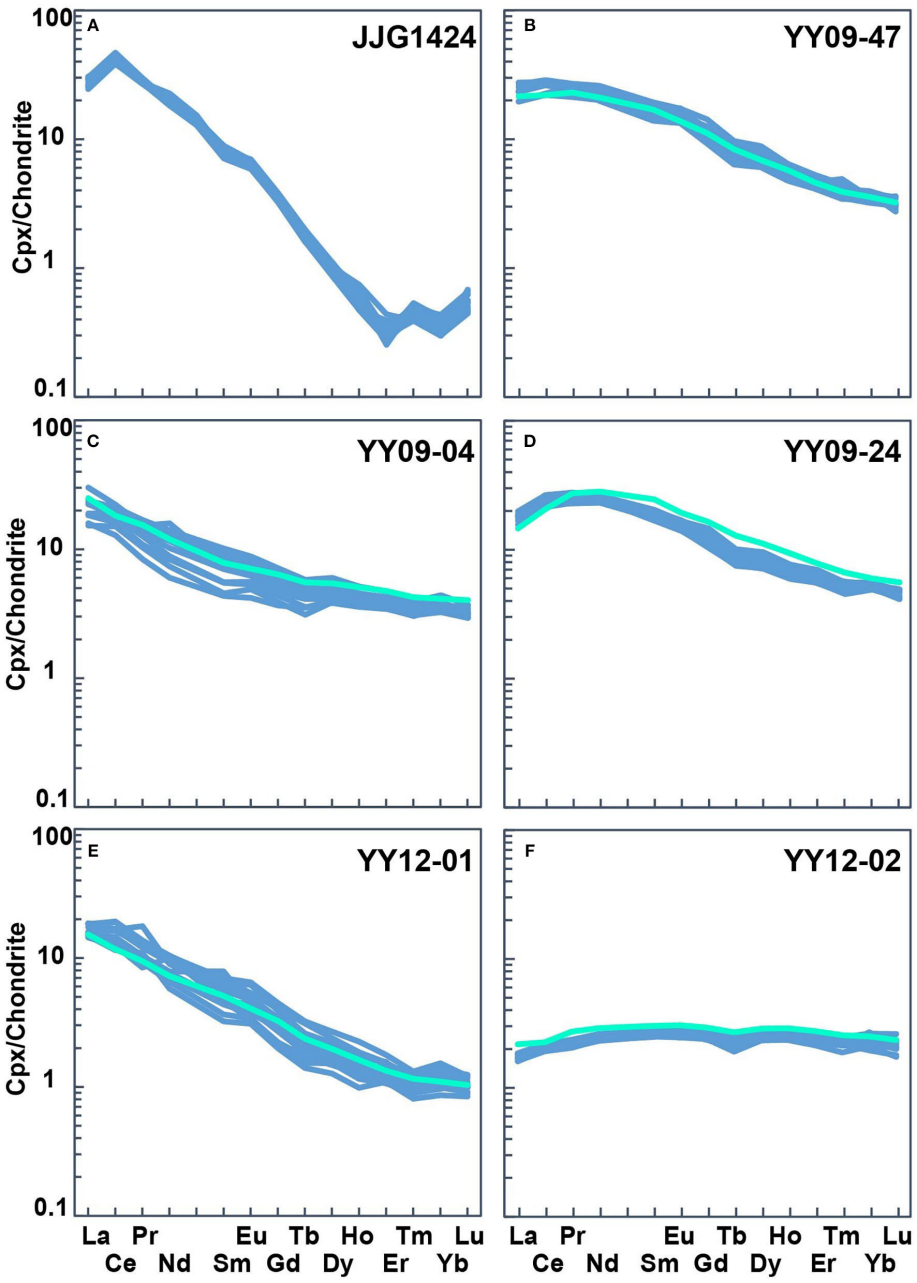
Rare earth element (REE) patterns of clinopyroxenes **(A–F)**, normalized to chondrite after (McDonough and Sun, 1995). The bright green lines are given by solution ICP-MS in **(B–F)** (Zhao et al., 2015), and the blue lines are measured by LA-ICP-MS.

**Figure 4:**
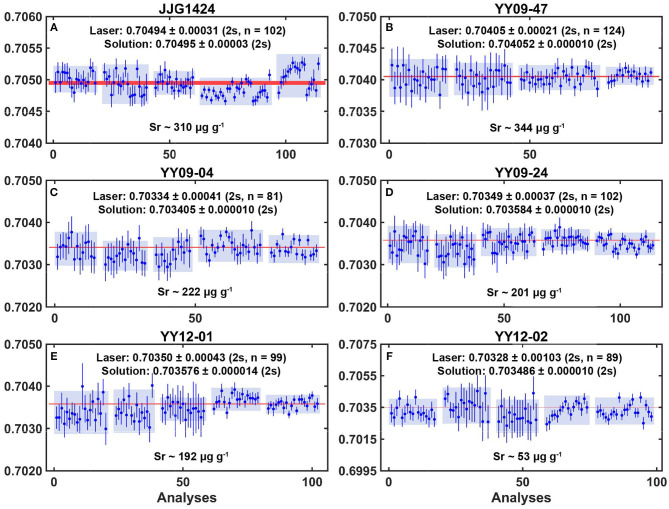
The measured ^87^Sr/^86^Sr ratios of six clinopyroxene samples over 2-year periods. The five light blue rectangle areas of each separate figure exhibit two standard deviation and subtraction on mean value of five sessions. The light red linear areas show reference values (for **A**, reference value from solution MC-ICP-MS by LeRoux et al., 2014; for **B–F**, reference values determined by ID-TIMS). The error bars are two standard deviations.

The grains generally appear to be structural homogeneous as indicated by backscattered microscopy (Figure 2). The average and ranges of the major and trace elements for these six clinopyroxenes are listed in Tables 2, 3 and displayed in Figure 3. Rb and Sr concentrations and Sr isotopic ratios are summarized in Tables 4, 5, Figure 4. To analyze Sr isotope ratios, especially for the samples with low Sr contents, the Rb/Sr ratio and Rb content do matter. The Rb/Sr ratios of clinopyroxene are normally below 0.01; thus, the radiogenic ^87^Sr contribution is insignificant (Yang et al., 2011, 2014, 2019; Tong et al., 2016; Zhang et al., 2018). In this research, the Rb/Sr ratios of samples fall in a range of 0.001–0.005.

**Table 2:** Major element (wt%) composition of the clinopyroxene investigated in this study.

**Sample**	**JJG1424**	**YY09-47**	**YY09-04**	**YY09-24**	**YY12-01**	**YY12-02**
SiO_2_	54.77	53.19	52.57	52.43	52.96	53.27
TiO_2_	0.25	0.12	0.36	0.46	0.34	0.21
Al_2_O_3_	2.91	5.69	5.62	6.67	6.72	5.13
Cr_2_O_3_	0.79	1.45	0.96	0.98	0.27	1.08
FeO	1.60	2.60	2.58	4.14	2.63	2.50
MnO	0.06	0.06	0.04	0.11	0.08	0.08
MgO	16.22	14.85	15.09	14.64	15.33	15.62
CaO	21.72	19.02	19.94	18.03	18.74	20.65
Na_2_O	1.67	2.28	1.60	1.90	2.02	1.33
NiO	0.02	0.04	0.04	0.07	-	-
Toal	100.03	99.30	98.80	99.50	99.20	99.90
Mg#	90.1	91.1	91.3	86.3	90.3	91.8
Cr#	10.5	14.6	10.3	8.9	2.6	12.4

**Table 3:** Trace element (μg g^−1^) composition of clinopyroxene measured by solution and laser ICP-MS.

	**JJG1424**	**YY09-47**		**YY09-04**		**YY09-24**		**YY12-01**		**YY12-02**	
	**Laser**	**Sol.**	**Laser**	**Sol.**	**Laser**	**Sol.**	**Laser**	**Sol.**	**Laser**	**Sol.**	**Laser**
La	18.0	13.7	15.2	15.8	13.3	9.19	11.3	9.81	10.5	1.40	1.11
Ce	68.2	36.3	43.8	29.8	30.0	33.7	39.9	19.5	25.1	3.77	3.47
Pr	7.10	5.77	6.57	3.81	3.34	6.84	6.28	2.39	2.89	0.70	0.56
Nd	24.5	25.9	28.1	14.5	13.2	34.7	30.2	8.99	10.6	3.68	3.09
Sm	3.25	6.71	6.59	3.05	2.74	9.78	7.22	2.05	2.17	1.25	1.09
Eu	0.98	2.05	2.25	1.04	0.96	2.90	2.24	0.62	0.69	0.48	0.42
Gd	1.88	5.80	6.00	3.31	2.75	8.66	6.20	1.76	1.68	1.62	1.46
Tb	0.18	0.84	0.80	0.56	0.45	1.32	0.87	0.25	0.23	0.29	0.24
Dy	0.67	4.36	4.64	3.49	2.93	7.21	5.20	1.32	1.28	1.97	1.79
Ho	0.08	0.80	0.78	0.72	0.59	1.34	0.94	0.24	0.23	0.44	0.38
Er	0.14	1.86	1.91	1.97	1.64	3.26	2.46	0.58	0.58	1.22	1.07
Tm	0.03	0.25	0.26	0.28	0.23	0.44	0.33	0.08	0.07	0.18	0.16
Yb	0.16	1.44	1.48	1.72	1.53	2.50	2.13	0.48	0.51	1.11	1.03
Lu	0.04	0.20	0.20	0.26	0.21	0.36	0.29	0.07	0.07	0.16	0.14
Y	1.88	20.4	20.9	17.3	15.3	32.1	24.1	6.31	5.87	11.6	9.70
Zr	17.7	63.7	34.0	82.6	40.5	171	68.4	33.1	24.6	17.5	7.43
Hf	1.50	1.03	0.90	1.57	1.22	2.29	2.07	0.73	0.88	0.57	0.38
Nb	–	1.80	0.86	3.11	2.14	3.21	1.93	1.52	0.82	1.34	0.49
Ta	–	0.09	0.06	0.23	0.16	0.43	0.25	0.12	0.11	0.07	0.05
Th	1.02	0.75	0.83	2.16	1.83	0.17	0.21	0.88	0.92	0.08	0.10
U	0.12	0.17	0.22	0.42	0.41	0.03	0.05	0.20	0.21	0.02	0.01
Rb	0.12	0.28	0.08	0.21	0.17	0.31	0.15	0.13	0.03	0.12	0.03
Ba	–	10.2	0.59	8.12	2.80	5.00	1.40	1.02	0.28	3.25	0.20
Sr	310	340	388	236	236	195	231	198	237	59.0	54.3
Ti	1379	763	1046	2272	1851	2256	1159	2022	642	1178	1552

**Table 4:** Summary of Sr isotopic ratios for six natural clinopyroxenes measured in five analytical sessions.

**Sample**	**Sessions**	**Date**	**Spot size (μm)**	**^**88**^Sr intensity (V)**	**^**84**^Sr/^**86**^Sr**	**2s**	**^**87**^Sr/^**86**^Sr**	**2s**	**n**	**MC-ICP-MS + Laser**	**Labs**
JJG1424	Session 1 Session 2 Session 3 Session 4 Session 5 **Mean**	2017.02.16 2017.05.06 2019.03.28 2019.07.06 2019.05.28	120 110 120 90 60	0.90 0.87 1.05 1.30 3.03 **1.62**	0.0570 0.0568 0.0569 0.0575 0.0578 **0.0573**	0.0010 0.0012 0.0010 0.0008 0.0006 **0.0012**	0.70501 0.70493 0.70495 0.70506 0.70481 **0.70494**	0.00021 0.00031 0.00019 0.00034 0.00022 **0.00031**	18 19 17 30 18 **102**	Neptune + Geolas Neptune Plus + G2 Neptune + Geolas Neptune Plus + Geolas Neptune Plus + NWR fs	IGGCAS IGGCAS IGGCAS IGGCAS CUG
YY09-47	Session 1 Session 2 Session 3 Session 4 Session 5 **Mean**	2017.02.16 2017.05.06 2019.03.28 2019.07.06 2019.05.28	120 110 120 90 60	0.79 0.69 1.08 1.34 2.29 **1.36**	0.0560 0.0557 0.0555 0.0564 0.0573 **0.0563**	0.0024 0.0018 0.0021 0.0010 0.0002 **0.0021**	0.70403 0.70401 0.70403 0.70406 0.70408 **0.70405**	0.00030 0.00026 0.00020 0.00013 0.00015 **0.00021**	20 20 28 21 35 **124**	Neptune + Geolas Neptune Plus + G2 Neptune + Geolas Neptune Plus + Geolas Neptune Plus + NWR fs	IGGCAS IGGCAS IGGCAS IGGCAS CUG
YY09-04	Session 1 Session 2 Session 3 Session 4 Session 5 **Mean**	2017.02.16 2017.05.06 2019.03.28 2019.07.06 2019.05.28	120 110 90 90 60	0.57 0.54 0.60 1.00 1.27 **0.83**	0.0568 0.0555 0.0551 0.0581 0.0575 **0.0568**	0.0016 0.0020 0.0024 0.0023 0.0003 **0.0030**	0.70338 0.70321 0.70324 0.70345 0.70337 **0.70334**	0.00040 0.00035 0.00041 0.00038 0.00033 **0.00041**	14 15 13 22 17 **81**	Neptune + Geolas Neptune Plus + G2 Neptune + Geolas Neptune Plus + Geolas Neptune Plus + NWR fs	IGGCAS IGGCAS IGGCAS IGGCAS CUG
YY09-24	Session 1 Session 2 Session 3 Session 4 Session 5 **Mean**	2017.02.16 2017.05.06 2019.03.28 2019.07.06 2019.05.28	120 110 90 90 60	0.57 0.52 0.63 0.76 1.37 **0.81**	0.0556 0.0552 0.0566 0.0569 0.0577 **0.0565**	0.0022 0.0022 0.0042 0.0012 0.0011 **0.0031**	0.70348 0.70333 0.70354 0.70359 0.70349 **0.70349**	0.00044 0.00039 0.00034 0.00025 0.00027 **0.00037**	17 17 23 20 25 **102**	Neptune + Geolas Neptune Plus + G2 Neptune + Geolas Neptune Plus + Geolas Neptune Plus + NWR fs	IGGCAS IGGCAS IGGCAS IGGCAS CUG
YY12-01	Session 1 Session 2 Session 3 Session 4 Session 5 **Mean**	2017.02.16 2017.05.06 2019.03.28 2019.07.06 2019.05.28	120 110 90 90 60	0.58 0.64 0.41 0.95 1.67 **0.87**	0.0559 0.0564 0.0560 0.0578 0.0578 **0.0568**	0.0029 0.0026 0.0032 0.0012 0.0006 **0.0029**	0.70338 0.70340 0.70348 0.70369 0.70358 **0.70358**	0.00051 0.00051 0.00028 0.00027 0.00022 **0.00043**	20 15 16 18 20 **99**	Neptune + Geolas Neptune Plus + G2 Neptune + Geolas Neptune Plus + Geolas Neptune Plus + NWR fs	IGGCAS IGGCAS IGGCAS IGGCAS CUG
YY12-02	Session 1 Session 2 Session 3 Session 4 Session 5 **Mean**	2017.02.16 2017.05.06 2019.03.28 2019.07.06 2019.05.28	150 130 110 90 60	0.16 0.16 0.12 0.34 0.49 **0.27**	0.0542 0.0522 0.0548 0.0540 0.0622 **0.0561**	0.0086 0.0074 0.0069 0.0037 0.0007 **0.0106**	0.70321 0.70361 0.70295 0.70332 0.70327 **0.70328**	0.00082 0.00115 0.00105 0.00093 0.00086 **0.00103**	17 17 15 16 21 **89**	Neptune + Geolas Neptune Plus + G2 Neptune + Geolas Neptune Plus + Geolas Neptune Plus + NWR fs	IGGCAS IGGCAS IGGCAS IGGCAS CUG

*NWR, a 257 nm femtosecond laser ablation system. Session 5 were performed at the State Key Laboratory of Geological Process and Mineral Resources (GPMR), China University of Geosciences (Wuhan), China. Bold values indicates the mean values for every sample above*.

**Table 5:** Comparison of Rb, Sr content and ^87^Sr/^86^Sr ratios of clinopyroxene using ID-TIIMS and LA-(MC)-ICP-MS.

**Sample**	**Rb (μg g^**−1**^)**	**Sr (μg g^**−1**^)**	**^**87**^Sr/^**86**^Sr**	**2s**	***n***	**Methods**
JJG1424^*^	0.16	309.6	0.704950	0.000030	1	Solution
	0.12	310	0.70494	0.00031	102	Laser
YY09-47	0.20	344.2	0.704052	0.000010	1	Solution
	0.87	389	0.70405	0.00021	124	Laser
YY09-04	0.21	222.4	0.703405	0.000010	1	Solution
	3.33	236	0.70334	0.00041	81	Laser
YY09-24	0.30	200.6	0.703584	0.000010	1	Solution
	1.86	231	0.70349	0.00037	102	Laser
YY12-01	0.09	192.1	0.703576	0.000014	1	Solution
	0.66	237	0.70358	0.00043	99	Laser
YY12-02	0.09	52.6	0.703486	0.000010	1	Solution
	0.40	54	0.70328	0.00103	89	Laser

* *Means data comes from LeRoux et al. (2014) and was determined by solution MC-ICP-MS*.

### CPX With About 300 μg g^−1^ Sr Content (JJG1424 and YY09-47)

Samples JJG1424 and YY09-47 have Sr content of 309.6 and 344.2 μg g^−1^, respectively. They show narrow varieties of elemental ranges and have Rb/Sr ratio of 0.0015–0.0017 (Table 4). Approximately 120 analyses were performed on each sample in five sessions, with the signal intensity of the ^88^Sr between 0.7 and 3.0 V. Overall, the measured ^87^Sr/^86^Sr values for JJG1424 fell in the range of 0.70481 to 0.70506, and for YY09-47, from 0.70401 to 0.70408 under different instrumental conditions in five separate sessions (Table 4, Figures 4A,B). The stable isotope ^84^Sr/^86^Sr is 0.0573 ± 0.0012 (2SD, *n* = 102) for JJG1424 and 0.0563 ± 0.0021 (2SD, *n* = 124) for YY09-47, respectively. Multiple *in situ* analysis of these clinopyroxenes over a period of 2 years gave average ^87^Sr/^86^Sr = 0.70494 ± 0.00031 (2SD, *n* = 102) and ^87^Sr/^86^Sr = 0.70405 ± 0.00021 (2SD, *n* = 124), in good agreement with the result from MC-ICP-MS solution analysis (0.70495 ± 0.00003, 2σ, *n* = 3, LeRoux et al., 2014) and TIMS solution analysis (0.704052 ± 0.000010, 2σ, *n* = 1), respectively. Analytical 2σ uncertainties during sessions 1–5 in each session were between 0.00013 and 0.00034 (Table 5).

### CPX With About 200 μg g^−1^ Sr Contents (YY09-04, YY09-24, and YY12-01)

Samples YY09-04, YY09-24, and YY12-01 have about 200 μg g^−1^ Sr (i.e., 222.4, 200.6, 192.1 μg g^−1^). They show narrow varieties of element ranges and have Rb/Sr ratios under 0.0015 (Table 4). About 100 analyses were performed on each sample in session 1, 2, 3, 4, and 5, totally, with the intensity of the ^88^Sr signal between 0.4 and 1.7 V. Overall, the measured ^87^Sr/^86^Sr values for YY09-04 fluctuated from 0.70321 to 0.70345; for YY09-24, from 0.70333 to 0.70359; and for YY12-01, from 0.70338 to 0.70369 under different instrumental conditions in five separate sessions (Table 4, Figures 4C,D,F). The stable isotope ^84^Sr/^86^Sr is 0.0568 ± 0.0030 (2SD, *n* = 81), 0.0565 ± 0.0031 (2SD, *n* = 102) and 0.0568 ± 0.0029 (2SD, *n* = 99), respectively. Multiple *in situ* analyses of these clinopyroxenes obtained over 2 years gave average values of ^87^Sr/^86^Sr = 0.70334 ± 0.00041 (2SD, *n* = 81) for YY09-04, ^87^Sr/^86^Sr = 0.70349 ± 0.00037 (2SD, *n* = 102) for YY09-24, and ^87^Sr/^86^Sr = 0.70358 ± 0.00043 (2SD, *n* = 99) for YY12-01, which matched well with the result from solution analysis (0.703405 ± 0.000010, 2σ, *n* = 1; 0.703584 ± 0.000010, 2σ, *n* = 1; 0.703576 ± 0.000014, 2σ, *n* = 1). Analytical 2σ uncertainties during session 1–5 in each session were between 0.00025 and 0.00051 (Table 5).

### CPX With About 50 μg g^−1^ Sr Contents (YY12-02)

Sample YY12-02 has the lowest Sr content (52.6 μg g^−1^) of the six samples and a Rb/Sr ratio of 0.0017 (Table 4). Seventeen, 17, 15, 16, and 21 measurements were performed on the sample in sessions 1, 2, 3, 4, and 5, respectively, with the signal intensity of the ^88^Sr between 0.15 and 0.5 V. In total, the measured ^87^Sr/^86^Sr values for YY12-02 ranged from 0.70295 to 0.70361 under different instrumental conditions in five separate sessions (Table 4, Figure 4F). The stable isotope ^84^Sr/^86^Sr is 0.0561 ± 0.0106 (2SD, *n* = 89). Multiple *in situ* analysis over a period of 2 years gave mean ^87^Sr/^86^Sr = 0.70328 ± 0.00103 (2SD, *n* = 89), which marginally falls within errors of the result from solution analysis (0.703486 ± 0.000010, 2σ, *n* = 1). Analytical 2σ uncertainties during session 1–5 in each session were between 0.00082 and 0.00115 (Table 5).

### *In situ* Sr Isotopic Measurement of Low Sr Content Clinopyroxene

**Figure 5:**
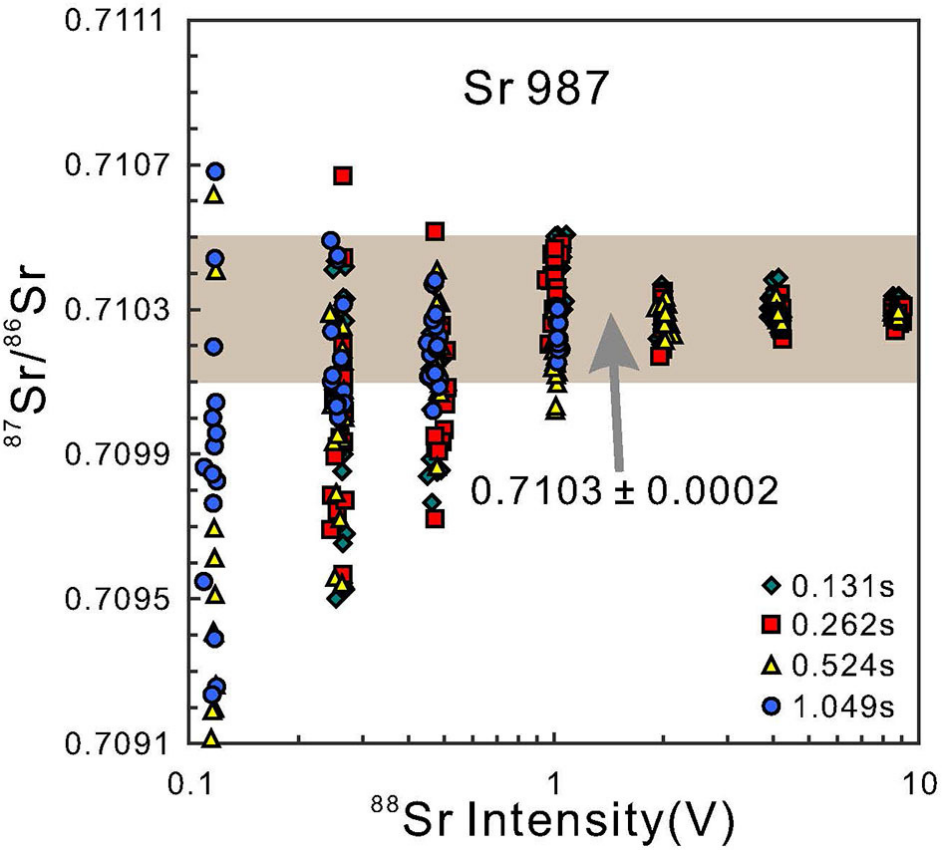
The relationship between the variation of ^87^Sr/^86^Sr ratios of NBS 987 and ^88^Sr signal intensity (volt) with different integration times, which simulate for low Sr content clinopyroxene sample under the similar laser mode and investigate the effect on accuracy and precision of low signal intensity using a Faraday cup. The gray rectangle means the accepted value of 0.7103 ± 0.0002 (2SD). The error bar is smaller than the symbol labels. As indicated, crater laser mode time can yield reasonable Sr isotope with of ± 0.0002 on the ^87^Sr/^86^Sr ratio, for the moderate Sr contents of clinopyroxene (more than 200 ppm Sr). However, a raster laser mode together with more integration time is preferable and desirable over the crater mode because of the relatively low Sr content samples (ca. 100 ppm Sr). Moreover, the ideal ^88^Sr signal intensity is more than ca. 1 V for reliable ^87^Sr/^86^Sr data.

As mentioned above, it is still challenging when it comes to reliably determining Sr isotope compositions of samples with low Sr content of <100 μg g^−1^ (Sun et al., 2012; Tong et al., 2016). While performing Sr isotopic measurements, sensitivities vary due to instrumental conditions and parameters. Figure 5 shows variations in ^87^Sr/^86^Sr results for analyses, under identical instrumental condition, of NBS 987 solutions at variable ^88^Sr signal intensity between 0.1 and 10 V. This suggests that, although the ^87^Sr/^86^Sr results deviate from the reference value as the signal drops, the longer integration time (1.049 s) can compensate and improve the precision. This improvement is especially pronounced when the intensity is lower than 0.5 V.

As summarized in Table 4, when the signal of the sample is lower than 1 V, particularly for samples with low Sr content (e.g., about 100 μg g^−1^ Sr). We utilized line scanning instead of single spot sampling in session 1 (120 μm spot size, nanosecond 193-nm laser) and session 5 (60 μm spot size, femtosecond 257-nm laser). Comparing with results of constant single point ablation, the ^88^Sr signal intensity lifted to 1 V, and the accuracy improved from 0.00051 (2SD, *n* = 20) to 0.00022 (2SD, *n* = 20). This reveals that we can get higher precision Sr isotopic data through nanosecond and femtosecond ablation by line scanning mode with moderate spot size even if the Sr content is <100 μg g^−1^. Therefore, for low Sr content samples, raster rather than crater laser mode, combined with more integration time, is preferable and can promote the precision and accuracy of actual low clinopyroxene sample. Moreover, the ideal ^88^Sr signal intensity is more than ca. 1 V for reliable ^87^Sr/^86^Sr data (Waight et al., 2002; Vroon et al., 2008; Jochum et al., 2009; Yang et al., 2014, 2019; Tong et al., 2016; Zhang et al., 2018).

### The Potential of Clinopyroxene for *in situ* Sr Isotopic Analysis

As mentioned above, Figure 1 illustrates that over 65% (10,010/15,306) published clinopyroxenes (*n* = 15,306, Georoc) have more strontium than YY12-02 sample with 50 μg g^−1^ Sr content, more than 40% (6,077/15,306) clinopyroxene contain over 100 μg g^−1^ strontium, and over 90% (13,863/15,306) are <350 μg g^−1^. Thus, clinopyroxene is a mineral that has low Sr content, and the suite of clinopyroxene reference materials have wide applicability and can cover the Sr content of the majority of natural clinopyroxenes.

Low element contents and isobaric interferences precluded Sr isotopic analysis on some clinopyroxene samples via LA-MC-ICP-MS. Our previous study indicated that ~500 μg g^−1^ Sr is enough to obtain an absolute precision of ± 0.0001 on the ^87^Sr/^86^Sr ratio when using a large laser spot size (Yang et al., 2009b, 2014, 2019). The extremely low Rb contents (and hence very low Rb/Sr ratios) of clinopyroxene mean that isobaric interference of ^87^Rb on ^87^Sr is usually negligible and can be easily accounted for (Yang et al., 2011). At more moderate to low Sr content of clinopyroxene (200 μg g^−1^), there is usually enough Sr for reasonable Sr isotopic analysis (± 0.0002). In our experiences, the Sr content of clinopyroxene determines the signal insensitivity and is the major factor for reliable Sr isotopic measurement by LA-MC-ICP-MS of this mineral phase (Wu et al., 2010; Yang et al., 2014, 2019; Zhang et al., 2018).

Based on our *in situ* Sr isotopic analyses of six potential clinopyroxene samples during 2-year periods (Figure 4, Table 5), we suggest that clinopyroxene with moderate Sr content ranging from 100 to 350 μg g^−1^ (e.g., JJG1424, YY09-47, YY09-04, YY09-24, and YY12-01) have statistically significant stability and homogeneity, making them excellent potential candidates for Sr isotopic microanalyses. Meanwhile, YY12-02 has lower content of elemental Sr, and its ^87^Sr/^86^Sr varies slightly (but marginally within errors of TIMS value), therefore making it a potential clinopyroxene reference material for samples with low content of Sr (Waight et al., 2002; Vroon et al., 2008; Jochum et al., 2009; Yang et al., 2014, 2019; Tong et al., 2016; Zhang et al., 2020).

## Conclusions

Considering the few, or unavailable, natural clinopyroxene reference materials for Sr microanalysis, we investigated thoroughly the assessment by both laser and solution measurements of the Sr isotopic ratios of six potential natural clinopyroxene reference materials from South Africa and China. The Sr isotopic compositions obtained for these samples are all consistent with values obtained by solution methods [both MC-ICP-MS and isotope dilution TIMS (ID-TIMS)]. Moreover, the major and trace elements of these clinopyroxenes were also examined by EPMA as well as solution and laser ICP-MS. Due to the abundant supply of these natural samples and their homogeneous Sr isotopic compositions, these clinopyroxene samples (JJG1424, YY09-47, YY09-04, YY09-24, and YY12-01) might be potential reference materials for *in situ* LA-MC-ICP-MS Sr isotopic measurements, and YY12-02 is a potential material to monitor analysis quality for low-Sr samples. Our results demonstrate that these samples can be employed as reference material for *in situ* determination of Sr concentration and isotope composition using laser sampling. Based on our data, laser ablation can yield reasonable Sr isotope with 2σ precision of ± 0.0002 on the ^87^Sr/^86^Sr ratio for clinopyroxene with moderate Sr contents (more than 200 μg g^−1^ Sr). Moreover, our diverse investigation indicates that the raster laser mode is preferable over the crater mode when analyzing samples with relatively low Sr content (ca. 100 μg g^−1^ Sr). These reference materials are of sufficient amount and are available to the scientific community via contacting the corresponding author.

## Data Availability Statement

The raw data supporting the conclusions of this article will be made available by the authors, without undue reservation.

## Author Contributions

HZ is a research scholar working on this study as part of his doctoral thesis, executed main experiments (major, trace, and Sr isotopes), analyzed and compiled the findings, and written this original manuscript preparation. X-MZ provided the five YangYuan samples and conducted trace element using solution ICP-MS and Sr isotope data by ID-TIMS. PL provided the JJG1424 sample and totally went through the draft. WZ is in charge of fs-LA-MC-ICP-MS at GPMR, Wuhan, conducted instrumental tuning, and supervised the fs-LA-MC-ICP MS work of HZ. L-WX, CH, and S-TW helped with the initial screening of CPX samples potentially suitable for LA-ICP-MS and ID-TIMS. HW, J-HY, and F-YW reviewed the draft and gave insightful comments. Y-HY supervised the whole study from planning to execution and result analysis. All authors contributed to the article and approved the submitted version.

## Conflict of Interest

The authors declare that the research was conducted in the absence of any commercial or financial relationships that could be construed as a potential conflict of interest.
